# The clinical application of customized 3D-printed porous tantalum scaffolds combined with Masquelet’s induced membrane technique to reconstruct infective segmental femoral defect

**DOI:** 10.1186/s13018-022-03371-3

**Published:** 2022-11-05

**Authors:** Yipeng Wu, Xiangwen Shi, Shaoneng Zi, Mingjun Li, Suli Chen, Chaoqun Zhang, Yongqing Xu

**Affiliations:** 1 Kunming Medical University, No. 1168 Yu Hua Street, Kunming, 650000 People’s Republic of China; 2 Institute of Traumatology and Orthopedics, 920th Hospital of Joint Logistics Support Force, PLA, Kunming, 650000 People’s Republic of China

**Keywords:** 3D printed, Tantalum, Prosthesis, Scaffolds, Infection, Bone graft, Segmental bone defect, Masquelet’s induced membrane technique (MIMT)

## Abstract

**Purpose:**

This study mainly exams a novel treatment for infective segmental femoral defect, and we combined the 3D printed porous tantalum prosthesis and Masquelet’s induce membrane technique to reconstruct bone defect and discussed the clinical effect.

**Method:**

The clinical research included 9 observational cases series, as a permanently implantation, the customized 3D-printed scaffolds that connected with an anatomical plate was implanted into the bone defect segment after successful formation of induced membrane, the clinical effect was evaluated by radiological exams and Paley’s bone union criteria.

**Result:**

The personalized 3D-printed porous tantalum was, respectively, manufactured and used in 9 consecutive patients to reconstruct the infective segmental bone defect of femur, the mean defect length was 16.1 ± 2.8 cm, the mean length of follow-up was 16.9 ± 4.0 months, after 2 stage operation, there was no deep infections, refractures, sensorimotor disorder, vascular injury, ankylosis and recurrence of infection occurred in all cases. postoperative radiological exams shown stable internal fixation and osseointegration, and all these results were invariable during the follow-up time in all cases. All patients significantly obtained deformity correction and length of limb.

**Conclusion:**

The customized 3D-printed porous tantalum prosthesis was an acceptable alternative treatment to the autogenous or allograft bone graft, the combination of the two techniques could achieve satisfactory reconstruct to infective broad bone defect in femur when other biological techniques were not suitable.

## Introduction

Posttraumatic segmental bone defect still poses a great challenge to orthopedic surgeon in the setting of infection owing to complicated complications and long disease course, the anti-infection, stably fixation, bony union need to be taken into account [1]. Since 2000, Masquelet’s induced membrane technique (MIMT) [2] has shown great promise to reconstruct bone defect caused by trauma, tumor or infection [3], compared with illizarov bone transport which is also widely accepted as one of ideal method for infective bone loss, MIMT leads to continuous anti-infection, less bone union time and better functional outcome [4] as a result of two-stage technique that include debridement, antibiotic loaded cement spacer, and autogenous bone graft [5]. The advantage of Masquelet technique had led to a world‐wide increase in MIMT procedures over the past 2 decades [6–9]. However, some literatures reported the increased risk of treatment failure for defect over 80 cc in volume [10], bone nonunion, iliac wing fracture, hematoma and insufficient functional exercise are less likely to avoid in broad infective bone defect cases regardless of the management [11].

The 3D-printed implant emerged as a novel mean for MIMT to reconstruct broad bone loss, studies have shown positive results of implanting customized 3D-printed titanium cages with limited autograft and allogeneic cancellous bone after infection controlling by antibiotic loaded cement spacer in stage I [1]. As a substitution of simply bone graft, the 3D printed titanium prosthesis may improve the clinical efficacy by providing stable scaffold for osteogenesis [12]. However, in terms of material science, 3D-printed porous tantalum has been proved to be more similar to bone scaffold than titanium in both microstructural and biomechanical properties [13]. Although 3D-printed porous tantalum prosthesis have been used in clinical studies in the field of orthopedics, both the limb sparing surgery of osteosarcoma and reconstruction surgery for infected bone defect after knee arthroplasty, the personalized prosthesis shown satisfactory outcomes [10, 14], to our knowledge, there are less systematically literature report about clinical application of 3D-printed porous tantalum prosthesis for segmental infected bone defects caused by osteomyelitis. This manuscript reported the preliminary outcomes in clinical series using customized 3D-printed porous tantalum scaffolds combined with MIMT to reconstruct infective femur defect, the procedure included a first stage with antibiotic cement spacer follow debridement as Maquelet’s description [2], then the 3D-printed porous tantalum prosthesis bonded to a locking plate was implanted into the spacer. The primary purpose of this article was to evaluate clinical effect of using a relatively new porous scaffolds as an alternative filler for bone graft.

## Materials and methods

Two clinically experienced orthopedic surgeons assess the outcome based on postoperative radiological findings and fracture healing scores. This study was approved by the Human Research Ethics Committee of Hospital No. 920. The results of this treatment was based on bone union status, infection control, deformity correction and limb length discrepancy, the same as the Paley [15] principle.

### Selecting criteria

#### Exclusion criteria

Juvenile patients (age < 18 years); unwilling or unable to participate in this study; patients with systemic multiple organ dysfunction or long-term treatment with steroids, acquired immunodeficiency diseases; length of follow-up time < 6 months. Inclusion criteria: adult patient (age ≥ 18 years), post-traumatic infective femoral bone loss, the length of bone defect > 5 cm after debridement, consent to use 3D-printed porous tantalum to complete reconstruction. X-ray, CT scan and 3D image, MRI and isotope bone scanning were performed to evaluate the length of bone defect and infective range of soft tissue in all patients, laboratory test as blood routine, liver and kidney function, blood biochemistry to access general condition, C reactive protein (CRP) and erythrocyte sedimentation rate (ESR) to estimate activity of inflammation. Meanwhile, some secretion samples from sinus tract were collected to perform bacterial cultivate and antibiotic sensitivity test in severe infective cases.

### Surgical procedure

All patients with infective femoral segmental bone defect underwent 2 steps operative technique as part of the treatment plan, the initial step involve resection of devitalized bone fragment and soft tissue according to preoperative radiological examination, the symbol of a successful debridement was fresh blood flowing out from both the skeleton stumps and soft tissue, avoid to injury vital vessels and nerves in deep tissue. The bone cavity was filled with vancomycin loaded cement spacer, stabilized by anatomical plate to maintain the length of femur and limb mobilization. The infective sample around bone fragment was collected again to confirm sensitive antibiotics. In this step, 4 g of vancomycin mixed with every 40 g of bone cement, the high-dose of antibiotics would release in local to control possibly remaining inflammation [11]. Patients kept on protected weight bearing before the next surgery, the debridement should performed again if infection recurrent.

The customized 3D-printed porous tantalum prosthesis was designed refer to the bone defect length and diameter of imaging results. The porous tantalum with an intramedullary structure firmly attached to a locking plate together to form a complete scaffold, and we restricted the porous diameter and porosity, respectively, in 350 μm and 70% to guarantee biomechanical stability. During the operation, cancellous bone was not implanted in the prosthesis. 4–6 months after the first stage, split the autologous membrane longitudinally to remove all bone cement spacer without residual, implanted the prosthesis after repeated flushing, for the purpose of confirmed fixation, at least 4 screws were inserted along the plater holes at both ends of the femur cortex, then the membrane was carefully sutured to form a biological milieu, and kept an negative pressure drainage tube close to the wound for 7–14 days, used antibiotics intravenously for 2 weeks according to the result of sensitive drug test. The limb motion exercise began immediately but avoid totally weight bearing practice within 1 month, partial weight bearing began from the second month with the assistance in crutches, all patients were required with radiological exams.

### Statistical analysis

For data conforming to a normal distribution, the mean ± standard deviation (SD) was used to express the data, and for non-normally distributed data, the median (25% percentile, 75% percentile) was used. The student’s t-test was used for normally distributed data, and the chi-square test was used for non-normally distributed data. *P* < 0.05 was considered statistically significant.

## Results

This study was a single-center retrospective study and composed of 9 clinical cases, included 6 males and 3 females. Infective bone defect area was, respectively, located at distal and proximal section of femur, bone defect length range was 10.2–19.8 cm, average 16.1 ± 2.8 cm. All cases presented segmental bone loss approach to hip or knee joint, the distance was 5.9–9.0 cm, average 7.1 ± 1.1 cm. According to preoperative measurement, 2 cases shown 3.5–5 degrees valgus and 7 cases existed 3.5–15.0 degree varus deformity, the mean length discrepancy was 0.7–8.0 cm, average 3.1 ± 2.3 cm compared with the contralateral limb, the mean follow-up time were 16.9 ± 4.0 months (Tables 1 and 2).

**Table 1:** The clinical data of patients

Case	Gender	Age (years)	Side	Original fixation	Bone defect location	Follow-up (months)	Coronal deformity (degree)	Limb length discrepancy (cm)
1	Male	23	Left	Internal fixation	Proximal femur	15	5.0 Valgus	0.7
2	Female	26	Left	external fixation	distal femur	13	5.5 Varous	1.5
3	Male	45	Right	Internal fixation	distal femur	12	6.5 Varous	4.8
4	Male	37	Right	external fixation	distal femur	17	4.8 Varous	3.7
5	Male	56	Left	Internal fixation	distal femur	20	3.5 Valgus	1.9
6	Female	43	Right	External fixation	distal femur	24	6.0 Varous	4.0
7	Male	60	Left	Internal fixation	Proximal femur	14	15.0 Valgus	8.0
8	Male	28	Right	External fixation	distal femur	21	3.6 Varous	1.5
9	Female	39	Right	Internal fixation	Proximal femur	16	4.5 Varous	1.7
Mean	/	39.7 ± 12.9	/	/	/	16.9 ± 4.0	5.5 (4.05, 5.75)	3.1 ± 2.3

**Table 2:** Bone defect data and treatment outcome

Case	Bone defect length (cm)	Distance to greater trochanter or fossa intercondyliodea (cm)	Time between stages (months)	Coronal deformity (degree)	Limb length discrepancy (cm)	Weight bearing time (weeks)
1	18.4	7.8	4	1.0 Valgus	0	10
2	15.9	6.5	6	3.0 Varous	0.5	8
3	17.5	8.2	6	1.9 Varous	1.0	16
4	14.3	6.1	4.5	2.5 Varous	0.7	12
5	10.2	5.9	4	3.0 Valgus	0.5	6
6	15.6	7.0	6	2.7 Varous	0.8	10
7	19.8	6.3	4	1.5 Valgus	2.7	15
8	16.0	9.0	4.5	1.3 Varous	0	6
9	17.4	6.8	5.5	1.6 Varous	0.4	8
Mean	16.1 ± 2.8	7.1 ± 1.1	4.5 (4.0, 6.0)	2.1 ± 0.76	0.5 (0.2, 0.9)	10.0 (7.0, 13.5)

**Fig. 1:**
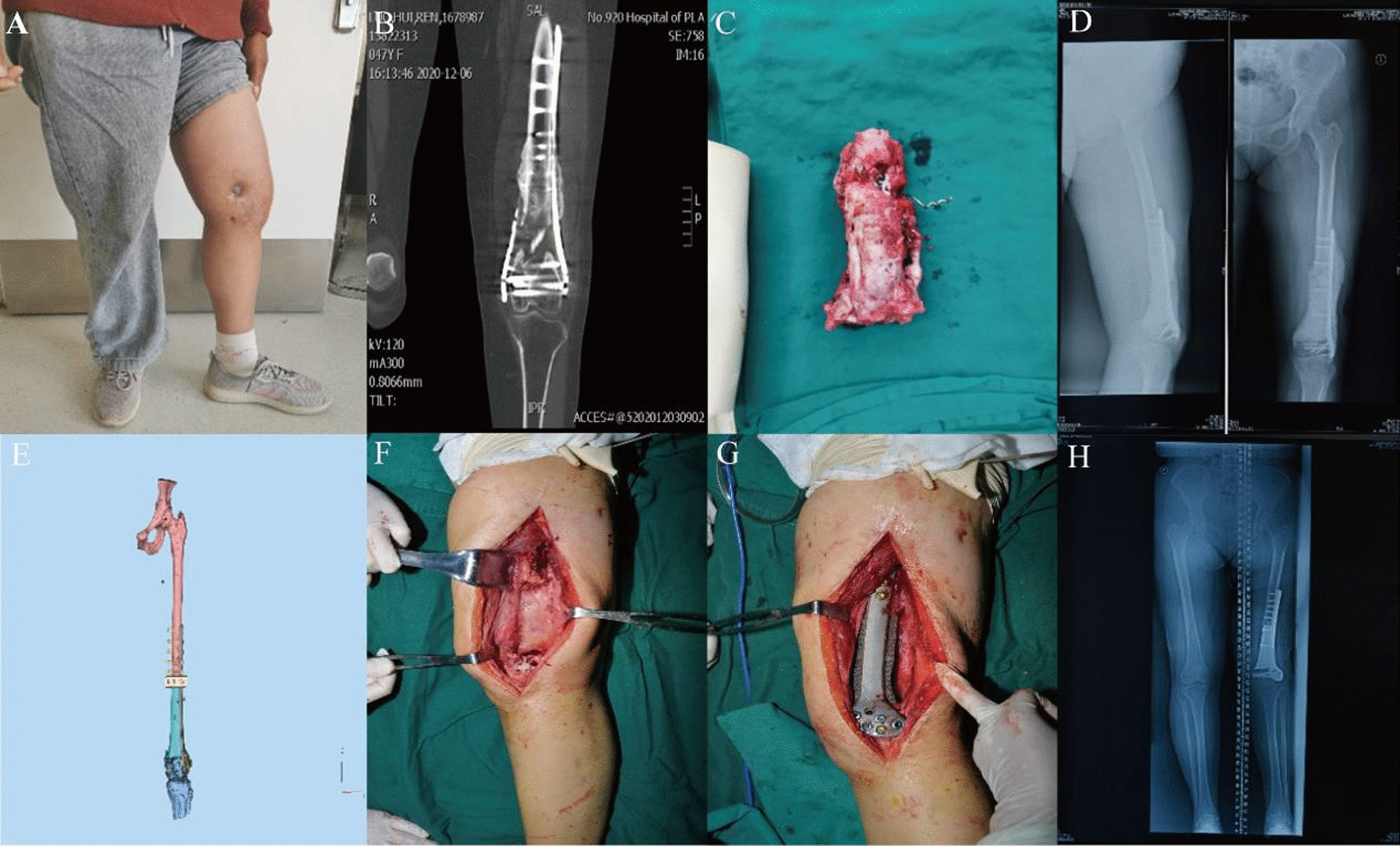
A typical case. **A** A 26-year-old female who suffered from comminuted femoral fracture in the distal section, two anatomical plates were used in the preliminary surgery, osteomyelitis occurred 3 months later with a sinus tract formation at inner side of thigh; **B** CT scan shows infective osteonecrosis in the distal section of left femur; **C** Removed the internal fixation and necrosis bone, the length of infective bone was approximate 15.9 cm; **D** Antibiotics-loaded bone cement spacer implanted into the defect section after debridement, combined with temporary fixation by a locking titanium plate on the lateral side of femur; **E** the photograph of real product, the prominent structure could insert into the medullary cavity to increase the contact area between the bone and prosthesis. **F** In the stage 2, remove all implantation in stage 1 and protected the self-induced membrane; **G** filled the segmental bone defect area with the scaffolds; **H** 13 months later, radiological tests showed satisfactory osseointegration of the scaffolds and stability fixation

**Fig. 2:**
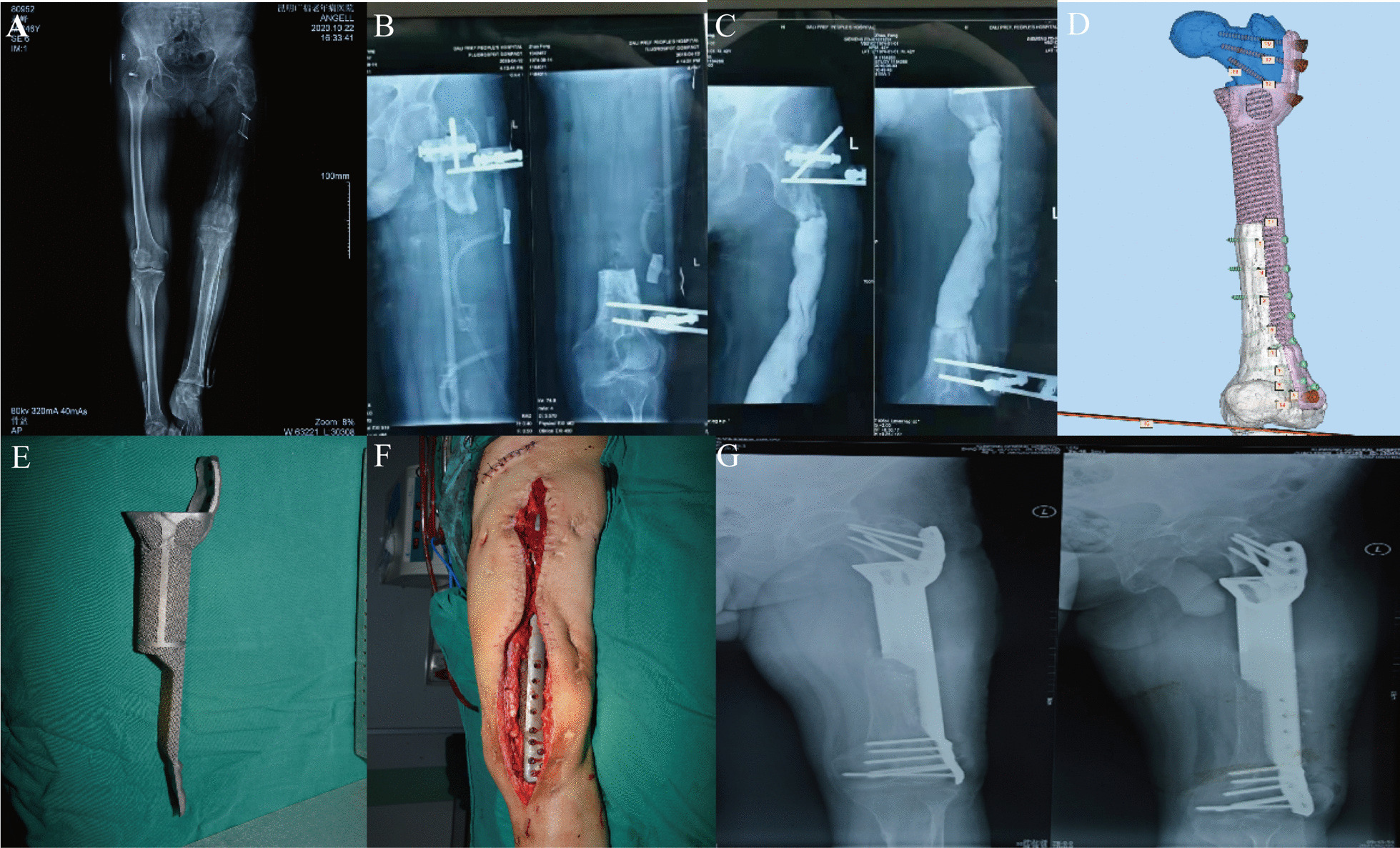
**A** A 60-year-old male with infective bone union on the left femoral, compared with the right-side limb, the patient presented serve varous deformity and length discrepancy. **B** The resection of infective necrosis bone was performed, used outfixation to sustain stability of the remained femur. **C** The vancomycin loaded bone cement spacer was filled in the bone defect section. **D** The design image of the customized 3D-printed prosthesis. **E** The photograph of real products, highly consistent with the specific shape of bone defect section. **F** Well fixation of the prosthesis after removing the outfixation and anti-infection filler. **G** X-ray image showed acceptable result without internal fixator failure in the last follow-up

The wound healed without sinus formation and secreta in all patients, postoperative radiological exams demonstrated good prosthesis integration with the cortex and cancellous (Figs. 1 and 2), there were no infection recurrence and deep infection in all case, the mean interval time between the two stages operation was 4.9 months, the mean limb length discrepancy reduced to minimum 0 cm and maximum 2.7 cm, the average weight-bearing time was month, all patients showed normal gait without pain, claudication in follow-up, 1 case present joint dysfunction due to insufficient joint practice, and joint function returned to basically normal under the guidance and assist of rehabilitation physician. According to the Paley score, the curative effect was excellent in 8 cases, well in 1 case.

**Fig. 3:**
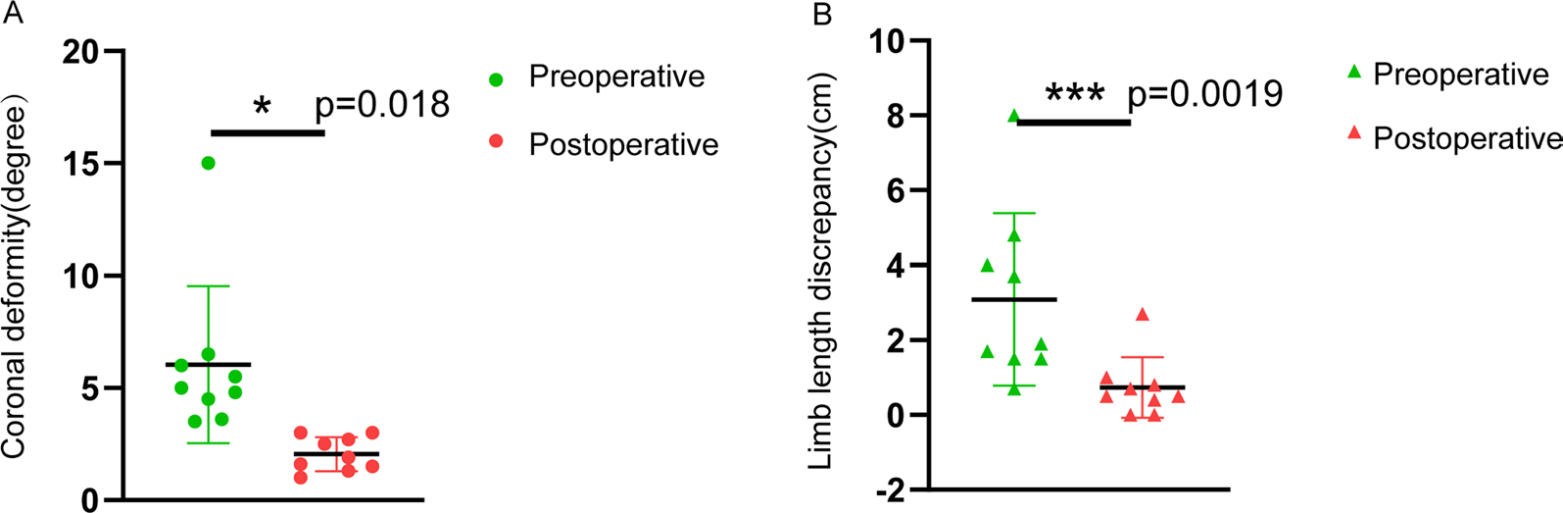
Analysis of the differences between preoperative and postoperative key indicators. **A** The overall coronal deformity angle in postoperative patients were significantly smaller than the preoperative coronal deformity angle (*P* = 0.018). **B** The overall Limb length discrepancy in postoperative patients were significantly smaller than the preoperative coronal deformity angle (*P* = 0.0019)

Importantly, the mean deformity angle of the patients changed from 5.5 (4.05, 5.75) degrees preoperatively to 2.1 ± 0.76 degrees postoperatively, with a significant reduction in deformity angle postoperatively (*P* = 0.018). The difference in mean limb length was significantly smaller in postoperative patients compared to preoperative (*P* = 0.0019) (Fig. 3).

## Discussion

Management for the segmental tubular bone defect often require appropriate treatment, prior literatures presented selectable strategy to reconstruct [16–19], most of their research shown satisfactory results and expectable abroad clinical application. In the condition of infection milieu, the illizarov bone transport and MIMT were acknowledged as the ideal management [4].

Although both the two techniques associate with uncertain bone union rates and long treatment time, surgeons often selected induced membrane technique as a priority owing to the in aesthetic appearance and inconvenience of illizarov external fixator [20], compared with traditional cylindrical bone transport fixator, the monoplane external fixator might means better tolerance for the patients during the lengthy bone tractive time, however, the deformity still appearance in the result of this substitute strategy [21], in addition, the technique was not suitable to reconstruct periarticular bone loss because of limited space to fix pins, and trans articular fixation may lead to stiffness and contracture of joint [4].

The MIMT with internal fixator seems to be solve the problems of illizarov bone transport, this alternative technique is also a feasible means to deal with infective bone defect of tubular bone and segmental bone defect adjacent joints [22], the main advantage is the immediately unrestricted range of motion practice after surgery which reduce the risk of myophagism, deep venous thrombosis and joint functional insufficiency [23], patients could achieve early functional exercise because the internal fixation and the autograft both contribute to establish a biomechanical stability. During first stage, the self-induced membrane is actually created by immune system, this characteristic combined with the loaded antibiotics will synergistically accomplish anti-infection in the process of bone reconstruction [3]. In the stage 2 of MIMT, segmental bone defect often requires huge volume of autologous and allogeneic bone which means enormous injury and may lead to chronic pain, fracture, hematoma and infection in the donor site [24].

In this study, we attempted to apply a novel means to improve the treatment in the second stage of MIMT, and, observed the stability of internal fixation and recovery of limb function, as a permanent implant, the postoperative imagine exam shown well osseointegration that no progressive relative movement between the implant and bone [25], through these indicators to infer the application prospect of 3D-printed porous tantalum. Previous study have already reported the 3D-printed titanium or tantalum prosthesis, with promising outcomes for the bone reconstruction, Li et al. [26] used a personalized 3D tantalum implant to reconstruct an approximately 12 × 8 × 6 cm bone defect after tumor removal, as an essential weight bearing skeleton of limb, there was no internal fixation failure and implant loosen present during the next 12 months. In the cases of bone necrosis and collapse, Mu et al. [27] applied customized talar prosthesis for arthroplasty after completely resection of talar, all cases achieved satisfactory results regarding pain relief and function recovery, a literature reviewed clinical result from revision surgery to oncologic reconstruction, indicated the good osseointegration, stably fixation and well biocompatibility of 3D-printed titanium or tantalum prosthesis [28].

The scaffold we designed was constructed with two parts, a porous prosthesis that made of tantalum was connected with a titanium locking plate, we choose porous tantalum instead of traditional titanium attributed to the better biomechanical and biological function of this metal, the material research conducted by Fan et al. [13] considered that cellular structure tantalum scaffold are superior to resist compression and deformation than titanium in similar construction. In an animal test, Ping et al. [29] concluded the tantalum with specific pore diameter and porosity has the biological ability to induce osteogenesis and osteointegration, prior study suggested the optimum porous diameter range was between 100 and 500 μm [30], additional research acknowledged the diameter of the pore in the porous structure greater than 300 μm promoted the formation of new bone and microcirculation [31]. Inheriting the above research, Li et al. [32] proceed a vitro experiment demonstrated that the porous diameter between 300 and 400 μm were more favorable to proliferation and osteogenic differentiation of the bone marrow stromal cells (BMSC). Therefore, in this study, we restricted the porous diameter and porosity, respectively, in 350 μm and 70%, to guarantee biomechanical stability and created a favorable osseointegration environment during the processes of bone repair.

Most of the infectious osteonecrosis in our study were located close to hip or knee joint and the minimum length of bone defect was 15 cm after debridement, all these cause great difficulty even for an experienced surgeon, the illizarov bone transport technique was the first excluded strategy as a result of considering the postoperative joint function [4], so MIMT was the ideal option. However, the bone graft in the stage 2 means abundant bone demand which may lead to multiple complication in the donor site [11]. Based on this consideration, we use the novel implant without bone graft in the stage 2, the length of the scaffolds could adjust according to specific length of bone defect in each case, another characteristic of the tantalum prosthesis is prominent structure allowed insert into the medullary cavity to partially enhance the connection with cancellous bone (Fig. 1). Furthermore, the whole implant was manufactured refer to the radiological exams, and the shape of the internal fixation was suitable to femoral anatomical characteristics. In open reduction surgery, this advantage indicates easier surgical procedure, less time and blood loss.

Although this study was preliminary clinical research, the result obviously shown encouragement outcomes, the limb deformity angle corrected from 5.5 (4.05, 5.75) degree preoperatively to 2.1 ± 0.76 degrees after surgery, without any changes by the last follow-up time, imagine test shown satisfactory osseointegration, Fig. 2 shown a special case with a length of femoral defect that approximately 20 cm. Moreover, both the remaining length of femur and morphology of the distal section posed great challenge for reconstruction, the illizarov and single MIMT technique was inappropriate to complete the re-establishment, and either traditional internal fixator could hardly enhance contact proportion with the cortex of femur [33], with the application of customized scaffolds and plate, the biomechanically stability was achieved and the prosthesis obtained enough contact proportion with the oblique fracture surface, which all benefit from the personalized shape of the implantation design [34]. However, the most meaningful limitation is the lack of comparison treatment group, and the selection bias may occur to influence the results of this research. Therefore, larger samples of this novel treatment and comparative studies with other means are required in the future.

This manuscript was not attempt to demonstrate that the 3D-printed porous tantalum scaffolds was omnipotent, in the treatment of weight-bearing long bone defection, each technology has advantage and drawbacks [4], the main purpose of our clinical research was trying to explore new path for bone reconstruction and offer more options for orthopedic surgeons.

## Conclusion

The study demonstrated the 3D-printed porous tantalum prosthesis combined with MIMT may achieve acceptable result in treating infective segmental femoral defect. The scaffold was most suitable for the broad periarticular bone defect on the account of joint functional protection, early joint practice and weight bearing, less complications on the donor site, despite this research still in the preliminary step, the scaffold recently showed acceptable outcomes as an alternative strategy to bone graft, means this technique have application potential in the future.

## Data Availability

All data and information supporting the article are available by contacting the corresponding author.
